# Supplementary material for: Ultrahigh pressure compaction-resistant thin film crosslinked composite reverse osmosis membranes
Source: Nat Commun. 2025 Sep 1;16:8165. doi: 10.1038/s41467-025-63639-0 (PMC12402197; doi:10.1038/s41467-025-63639-0)
Supplement: Supplementary file 1 — Supplementary Information [file 41467_2025_63639_MOESM1_ESM.pdf]

Ultrahigh Pressure Compaction-resistant Thin Film Crosslinked Composite Reverse Osmosis Membranes

Supplementary Information

Jishan Wu[1,2,†], Javier A. Quezada-Renteria[1,†], Jinlong He[3], Minhao Xiao[1], Yuanmiaoliang Chen[2], Hanqing Fan[2], Xinyi Wang[1], Fiona Chen[4],Kevin Pataroque[5], Yara Suleiman[6], Sina Shahbazmohamadi[6], Sreejith N.A.[7], Hariswaran Sitaraman[7], Marcus Day[7], Ying Li[8], David Jassby[1], Jeffrey R. McCutcheon[9], Menachem Elimelech[2,4], Eric M.V. Hoek[1,10,*]

[1] Department of Civil & Environmental Engineering, University of California, Los Angeles, CA 90095, United States
[2] Department of Civil & Environmental Engineering, Rice University, Houston, TX 77251, United States
[3] Failure Mechanics and Engineering Disaster Prevention Key Laboratory of Sichuan Province, Sichuan University, Chengdu 610207, China
[4] Department of Chemical & Biomolecular Engineering, Rice University, Houston, TX 77251, United States
[5] Department of Chemical & Environmental Engineering, Yale University, New Haven, CT 06511, United States
[6] Department of Biomedical Engineering, University of Connecticut, Storrs, CT 06030, United States
[7] Computational Science Center, National Renewable Energy Laboratory, 15013 Denver West Parkway, Golden, CO 80401, United States
[8] Department of Mechanical Engineering, University of Wisconsin-Madison, Madison, WI 53706, United States
[9] Department of Chemical & Biomolecular Engineering, University of Connecticut, Storrs, CT 06269, United States
[10] Energy Storage & Distributed Resources Division, Lawrence Berkeley National Lab, Berkeley, CA 94720, United States
† Jishan Wu and Javier A. Quezada-Renteria contribute equally to this work
* Corresponding author: emvhoek@ucla.edu (E.H.)

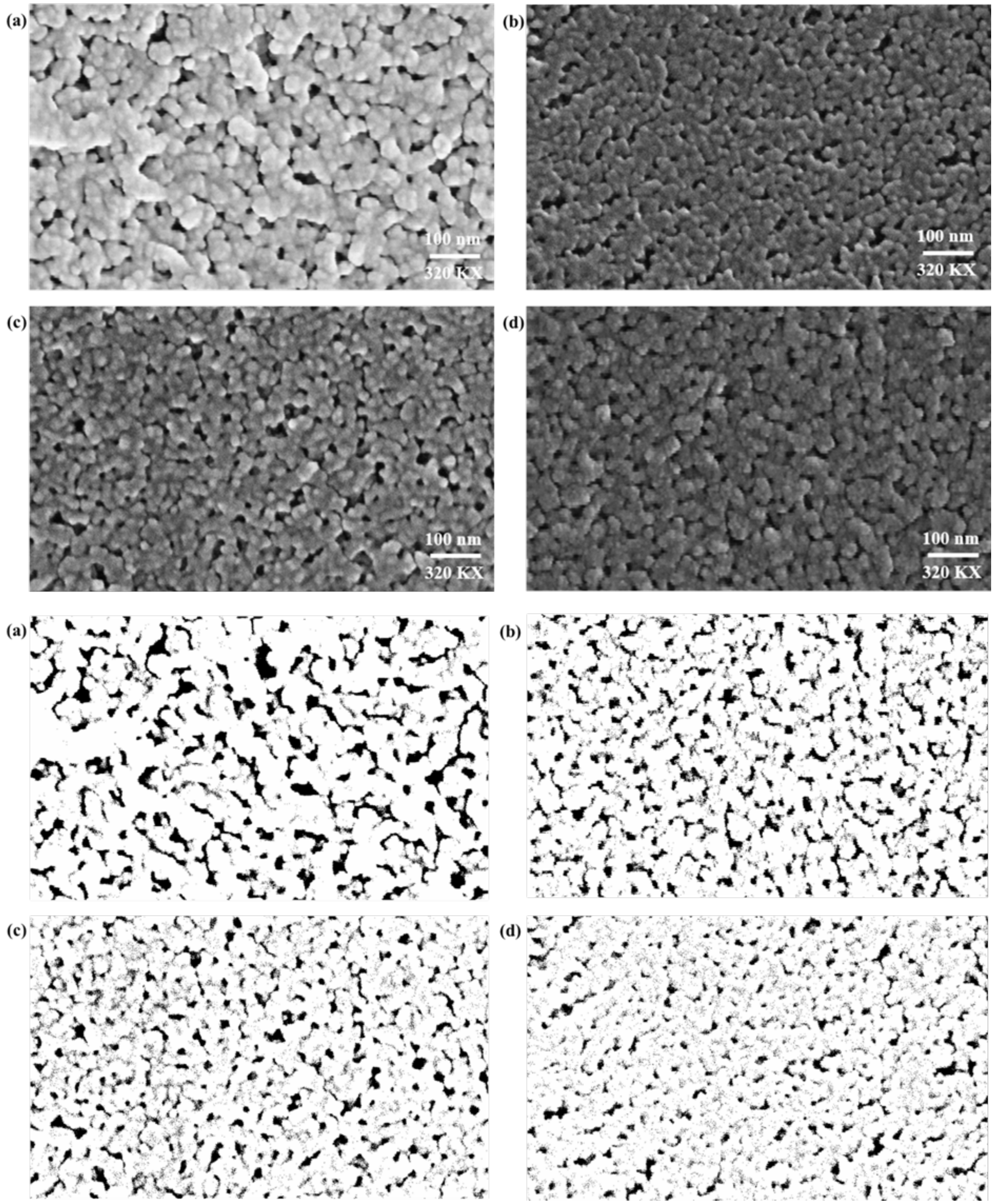

Fig. S1. SEM images and threshold processed images of (a) 16%, (b) 18%, (c) 20% and (d) 22% PI membranes.

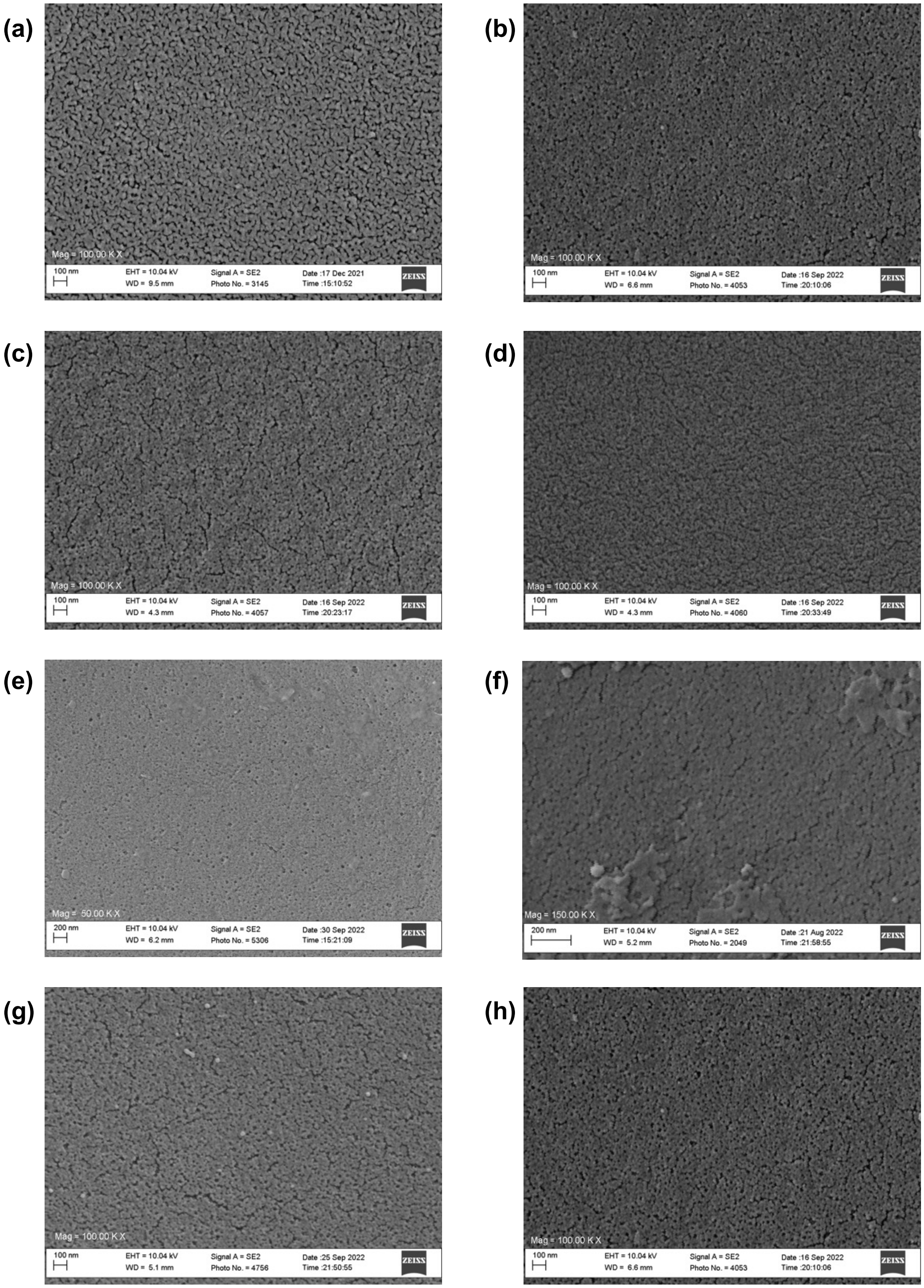

Fig. S2 Surface SEM images of casting solutions phase inverted into (a) DI water from 20% PI in DMF; (b) IPA from 20% PI in DMF; (c) IPA from 20% PI in 1:3 DMF:1,4-Dioxane;(d) IPA from 20% PI in 1:1 DMF:1,4-Dioxane; (e) DI from 20% PI in 1:3 DMF:1,4-Dioxane and *ex situ* crosslinked; (f) DI from 20% PI in 1:1 DMF:1,4-Dioxane and *ex situ* crosslinked; (g) IPA from 20% PI 1:3 DMF:1,4-Dioxane and *ex situ* crosslinked; (h) IPA from 20% PI in 1:1 DMF:1,4-Dioxane and *ex situ* crosslinked.

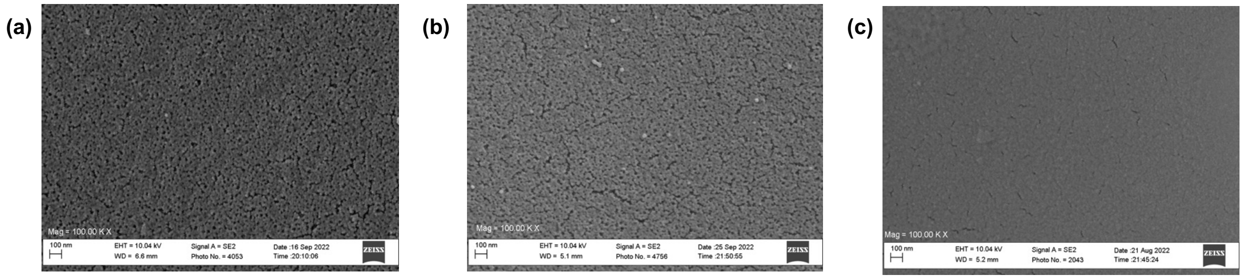

Fig. S3 SEM images comparing the surface morphologies of (a) 16% PI (DMF:1,4-Dioxane = 1:3) phase inverted in IPA; (b) 16% PI (DMF:1,4-Dioxane = 1:3) phase inverted in IPA and *ex situ* crosslinked; (c) 16% PI (DMF:1,4-Dioxane = 1:3) phase inverted in IPA and *insitu* crosslinked.

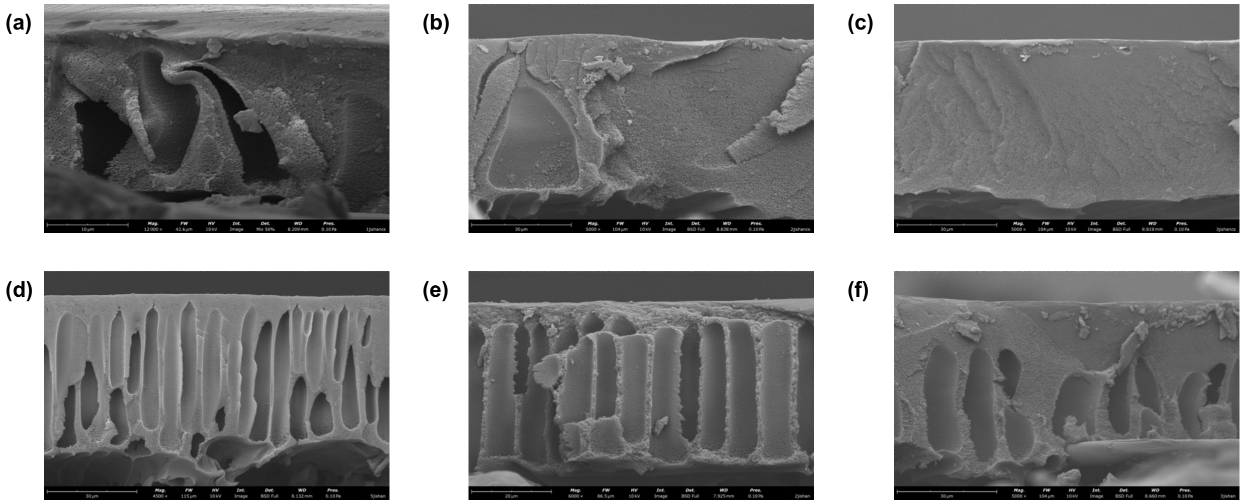

Fig. S4 Cross-sectonal SEM images of (a) 16% PI (DMF:1,4-Dioxane = 1:3) phase inverted in DI water; (b) 18% PI (DMF:1,4-Dioxane = 1:3) phase inverted in DI water; (c) 20% PI (DMF:1,4-Dioxane = 1:3) phase inverted in DI;(d) 16% PI (DMF:1,4-Dioxane = 1:1) phase inverted in DI water; (e) 18% PI (DMF:1,4-Dioxane = 1:1) phase inverted in DI water; (f) 20% PI (DMF:1,4-Dioxane = 1:1) phase inverted in DI water.

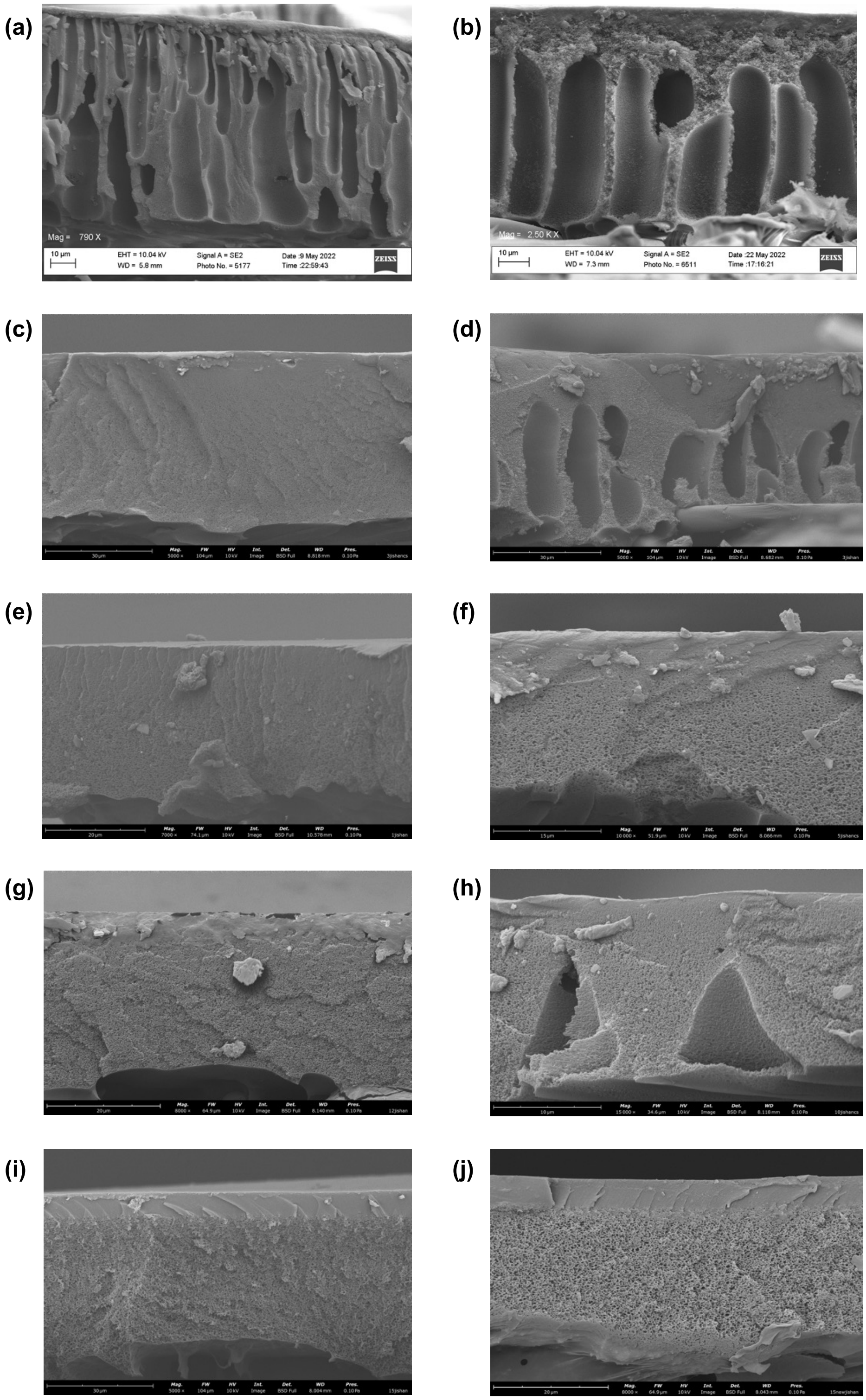

Fig. S5 Cross-sectonal SEM images of (a) 20% PI (DMF) phase inverted in DI water; (b) 20% PI (DMF) phase inverted in IPA; (c) 20% PI (DMF:1,4-Dioxane = 1:3) phase inverted in DI;(d) 20% PI (DMF:1,4-Dioxane = 1:1) phase inverted in DI; (e) 20% PI (DMF:1,4-Dioxane = 1:3) phase inverted in IPA; (f) 20% PI (DMF:1,4-Dioxane = 1:1) phase inverted in IPA; (g) 20% PI (DMF:1,4-Dioxane = 1:3) phase inverted in DI and *ex situ* crosslinked; (h) 20% PI (DMF:1,4-Dioxane = 1:1) phase inverted in DI and *ex situ* crosslinked; (i) 20% PI (DMF:1,4-Dioxane = 1:3) phase inverted in IPA and *ex situ* crosslinked; (j) 20% PI (DMF:1,4-Dioxane = 1:1) phase inverted in IPA and *ex situ* crosslinked.

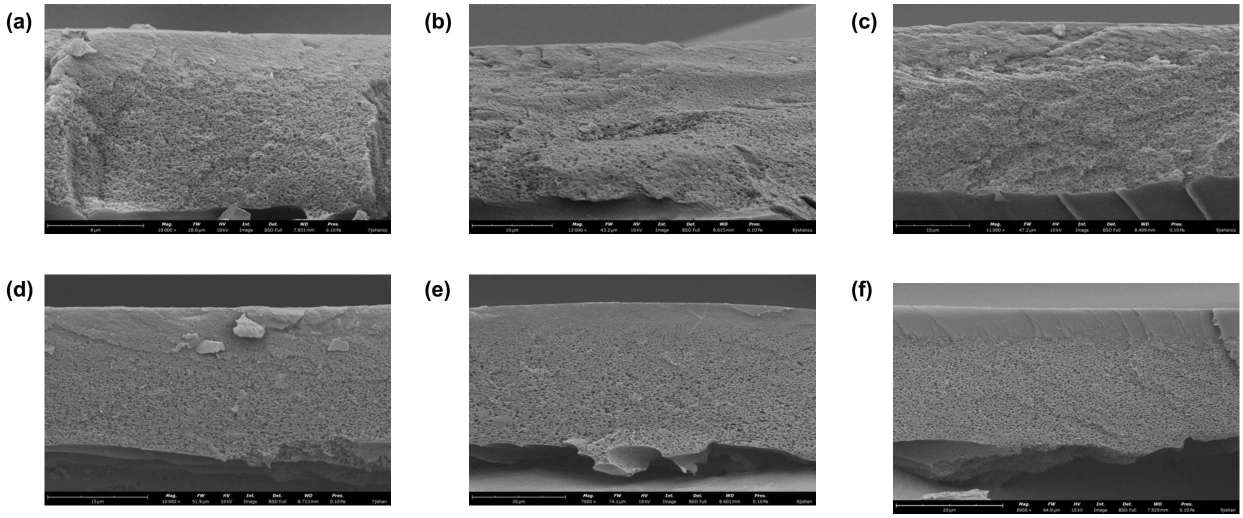

Fig. S6 Cross-sectional SEM images of of (a) 16% PI (DMF:1,4-Dioxane = 1:1) phase inverted and *insitu* crosslinked in HDA-IPA bath; (b) 18% PI (DMF:1,4-Dioxane = 1:1) phase inverted and *insitu* crosslinked in HDA-IPA bath; (c) 20% PI (DMF:1,4-Dioxane = 1:1) phase inverted and *insitu* crosslinked in HDA-IPA bath; (d) 16% PI (DMF:1,4-Dioxane = 1:3) phase inverted and *insitu* crosslinked in HDA-IPA bath; (e) 18% PI (DMF:1,4-Dioxane = 1:3) phase inverted and *insitu* crosslinked in HDA-IPA bath; (f) 20% PI (DMF:1,4-Dioxane = 1:3) phase inverted and *insitu* crosslinked in HDA-IPA bath.

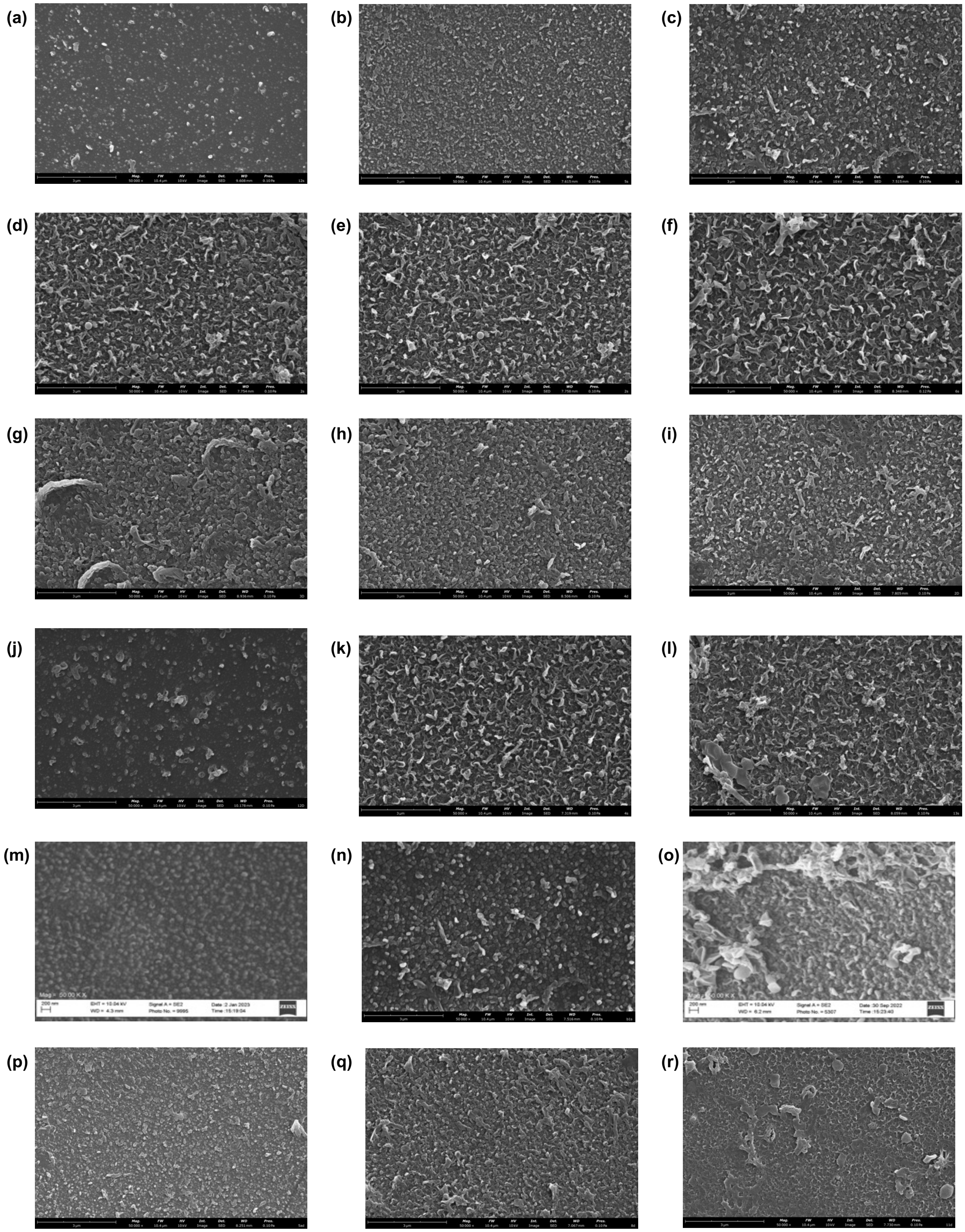

Fig. S7 Surface SEM images of TFX membranes. *ex situ* TFX membranes: (a) *e-TFX 1*; (b) *e-TFX 2*; (c) *e-TFX 3*; (d) *e-TFX 4*; (e) *e-TFX 5*; (f) *e-TFX 6*; (g) *e-TFX 7*; (h) *e-TFX 8*; (i) *e-TFX 9*; (j) *e-TFX 10*; (k) *e-TFX 11*; (l) *e-TFX 12*. *in situ* TFX membranes: (m) *i-TFX 1*; (n) *i-TFX 2*; (o) *i-TFX 3*; (p) *i-TFX 4*; (q) *i-TFX 5*; (r) *i-TFX 6*.

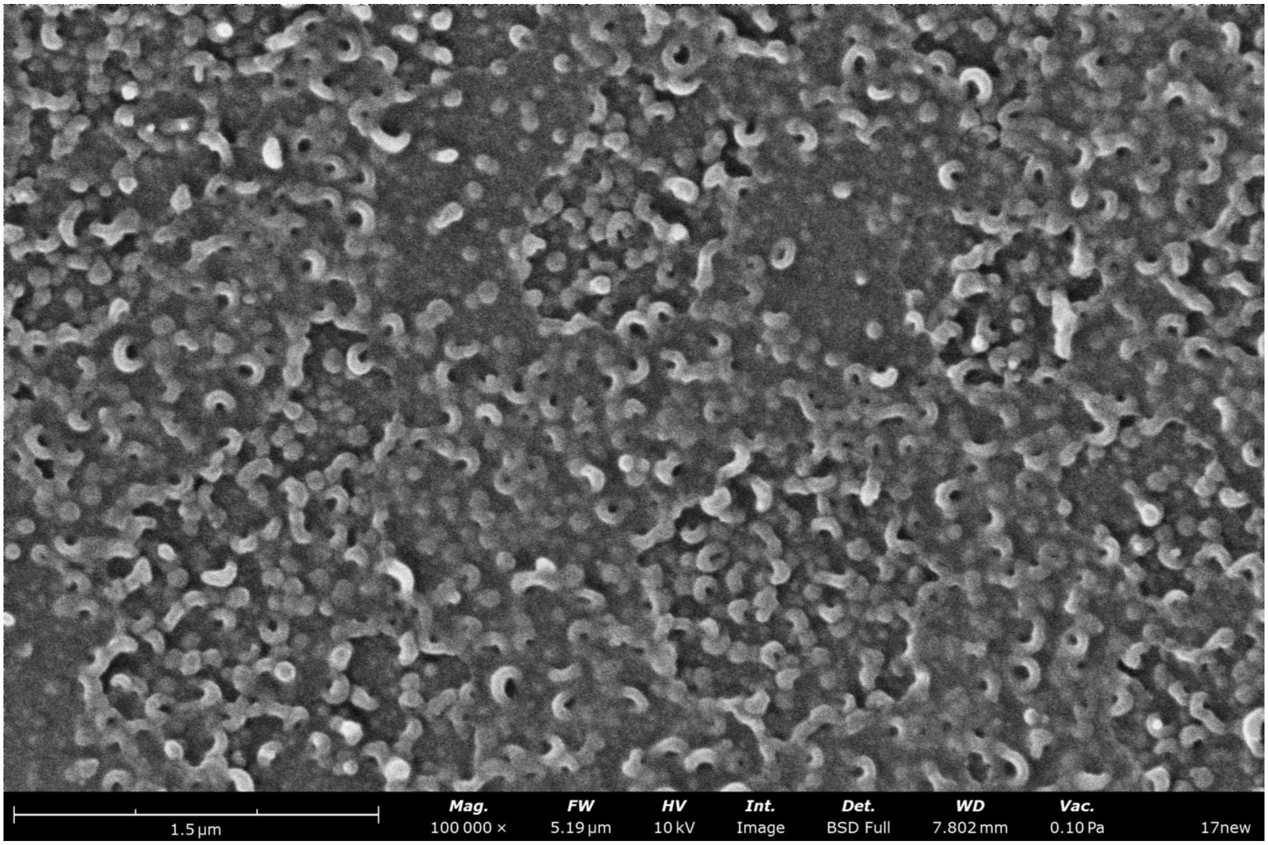

Fig. S8 Surface SEM images of *t-TFX* membranes.

(a) (b) (c)

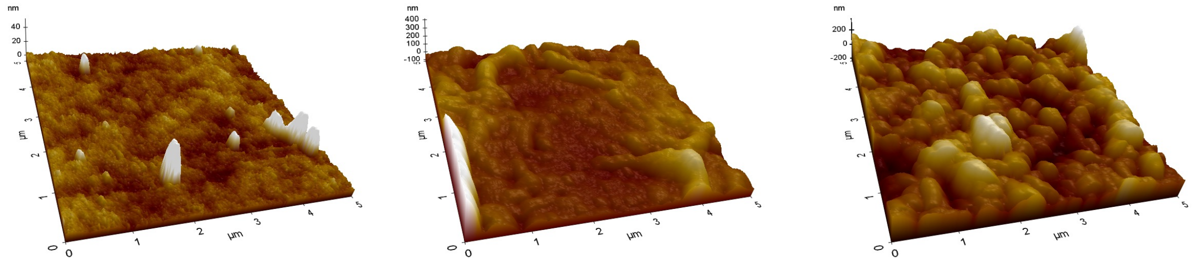

i-TFX	e-TFX	t-TFX
R_q = 64.6 nm	R_q = 81.2 nm	R_q = 114.7 nm
R_a = 51.7 nm	R_a = 64.9 nm	R_a = 91.76 nm
S_{sk}= 0.8	S_{sk}= 0.7	S_{sk}= 1.5

Fig. S9 AFM images and roughness measurements of TFX membranes. (a) *i-TFX*; (b) *e-TFX*; (c) *t-TFX.*

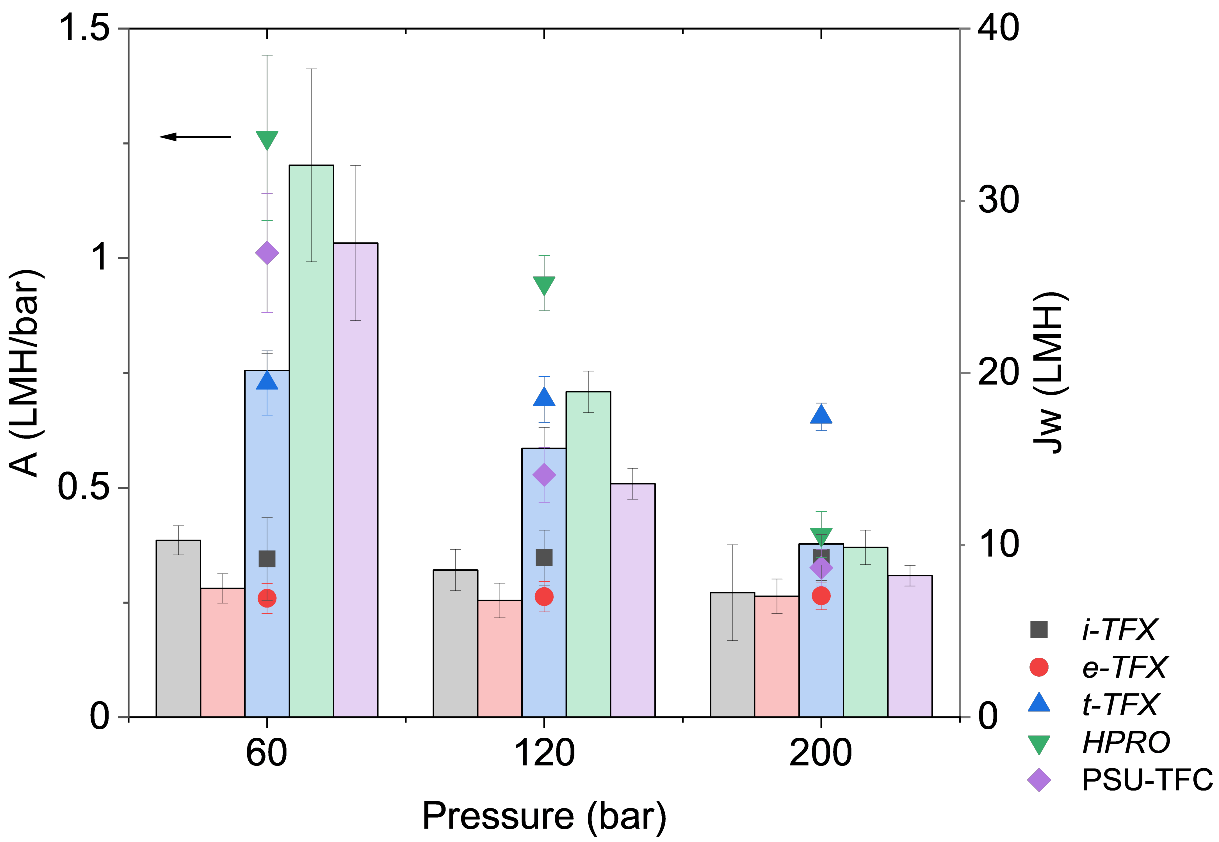

Fig. S10 TFX membrane versus commercial HPRO and handcast PSU-TFC membrane wet-testing performance. Water permeance is presented in LMH/bar (liter m^{-2} h^{-1} bar^{-1}) and flux is presented in LMH ((liter m^{-2} h^{-1}). Data represent the mean ± standard deviation from three independent experiments, where applicable.

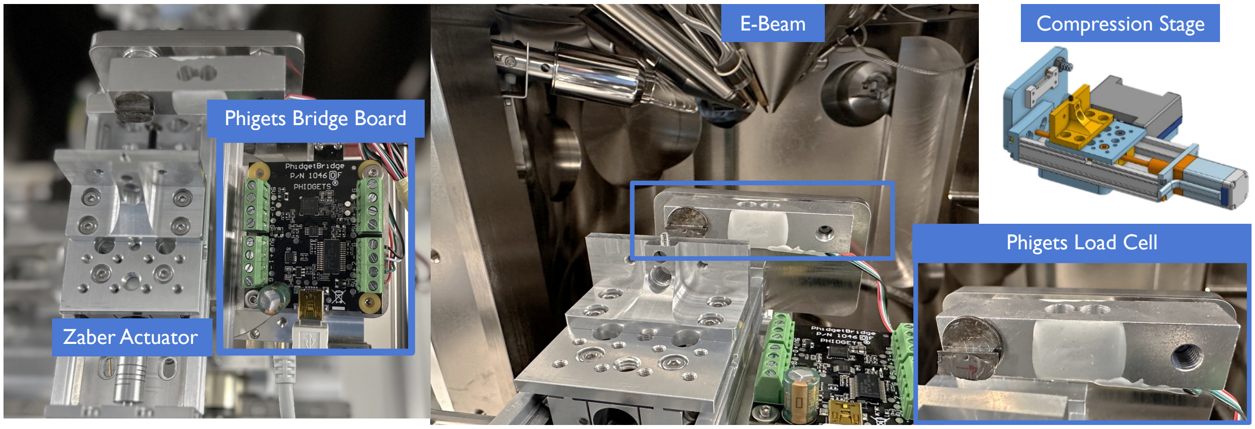

Fig. S11 *In operando* SEM imaging setup. The Zaber actuator provides precise control over the movement of the compression stage. The Phidgets load cell measures the force applied to the sample with high accuracy. The Phidgets bridge board connects the load cell to the control system, enabling signal processing and data acquisition. The electron beam (E-beam) enables real-time imaging. The in situ compression stage is customized for evaluating membrane compaction behavior.

Table S1. Contact angle of uncrosslinked and crosslinked PI membranes.

Membrane	Contact Angle (%)
16% NX	62.4 ± 2.2
18% NX	63.7 ± 1.3
20% NX	61.5 ± 1.1
22% NX	59.3 ± 2.7
18% *ex situ*	58.3 ± 0.4
18% *in situ*	57.1 ± 0.9
18% NX (DMF:1,4-Dioxane = 1:1)	62.4 ± 0.9
18% *ex situ* (DMF:1,4-Dioxane = 1:1)	55.9 ± 1.2
18% *in situ* (DMF:1,4-Dioxane = 1:1)	55.2 ± 0.8
18% NX (DMF:1,4-Dioxane = 1:2)	60.4 ± 0.8
18% *ex situ* (DMF:1,4-Dioxane = 1:2)	53.3 ± 0.5
18% *in situ* (DMF:1,4-Dioxane = 1:2)	54.8 ± 0.7
18% NX (DMF:1,4-Dioxane = 1:3)	53.9 ± 0.9
18% *ex situ* (DMF:1,4-Dioxane = 1:3)	47.3 ± 0.8
18% *in situ* (DMF:1,4-Dioxane = 1:3)	48.5 ± 0.7

Table S2. Testing performance of *ex situ* TFX membranes.

Membrane	Composition	Pressure (bar)	Jsw (lmh)	Rob (%)	A (lmh/bar)	B (lmh)
e-TFX 1	16% PI 3:1 IPA bath *Ex situ*	60	6.41	98.11	0.22	0.12
		120	5.96	96.93	0.21	0.18
		200	5.96	92.75	0.19	0.44
e-TFX 2	18% PI 3:1 IPA bath *Ex situ*	60	7.52	99.40	0.26	0.04
		120	6.74	97.70	0.26	0.15
		200	6.91	96.33	0.26	0.25
e-TFX 3	20% PI 3:1 IPA bath *Ex situ*	60	6.58	98.03	0.22	0.13
		120	6.37	96.23	0.20	0.24
		200	5.75	95.43	0.20	0.26
e-TFX 4	16% PI 1:1 IPA bath *Ex situ*	60	11.51	22.70	0.22	36.62
		120	10.89	22.72	0.11	34.70
		200	9.45	33.95	0.07	17.31
e-TFX 5	18% PI	60	9.04	75.38	0.25	2.78

	1:1 IPA bath *Ex situ*	120	8.63	52.95	0.13	7.24
		200	7.81	48.54	0.08	7.84
e-TFX 6	20% PI 1:1 IPA bath *Ex situ*	60	9.87	83.31	0.29	1.86
		120	9.87	55.37	0.15	7.47
		200	10.07	59.24	0.12	6.51
e-TFX 7	16% PI 3:1 DI bath *Ex situ*	60	14.80	49.38	0.49	13.65
		120	13.56	51.23	0.46	11.67
		200	11.30	59.17	0.42	7.11
e-TFX 8	18% PI 3:1 DI bath *Ex situ*	60	21.99	52.19	0.73	17.65
		120	19.73	58.81	0.69	12.20
		200	14.59	50.66	0.52	12.79
e-TFX 9	20% PI 3:1 DI bath *Ex situ*	60	8.84	93.81	0.30	0.54
		120	8.02	94.84	0.28	0.40
		200	6.58	88.08	0.26	0.82
e-TFX 10	16% PI 1:1 DI bath	60	19.73	55.75	0.63	13.83
		120	13.98	43.18	0.53	16.60

	Ex situ	200	12.33	36.12	0.44	19.80
e-TFX 11	18% PI 1:1 DI bath *Ex situ*	60	9.45	75.38	0.32	2.83
		120	8.84	52.94	0.30	7.22
		200	7.81	49.87	0.28	7.25
e-TFX 12	20% PI 1:1 DI bath *Ex situ*	60	10.48	69.22	0.36	4.26
		120	8.84	64.48	0.31	4.48
		200	6.99	44.32	0.28	8.13

Table S3. *In situ* TFX testing performance.

Membrane	Composition	Pressure (bar)	Jsw (lmh)	Rob (%)	A (lmh/bar)	B (lmh)
i-TFX 1	16% PI 3:1 HDA-IPA bath	60	13.15	85.15	0.40	2.13
		120	10.89	86.53	0.33	1.59
		200	9.37	72.33	0.16	3.37
i-TFX 2	18% PI 3:1 HDA-IPA bath	60	11.76	91.03	0.38	1.08
		120	9.87	89.71	0.31	1.06
		200	7.52	83.78	0.18	1.38
i-TFX 3	20% PI 3:1 HDA-IPA bath	60	10.69	98.87	0.35	0.11
		120	8.84	98.75	0.35	0.11
		200	6.99	98.40	0.35	0.11
i-TFX 4	16% PI 1:1 HDA-IPA bath	60	7.60	98.58	0.25	0.10
		120	6.78	98.90	0.25	0.07
		200	5.14	74.05	0.09	1.72
i-TFX 5	18% PI	60	3.29	97.05	0.11	0.06

	1:1 HDA-IPA bath	120	2.88	99.24	0.10	0.03
		200	2.88	99.05	0.10	0.03
i-TFX 6	20% PI 1:1 HDA-IPA bath	60	2.88	99.14	0.09	0.02
		120	2.67	98.84	0.09	0.03
		200	2.47	98.40	0.09	0.04

Table S4. Non-crosslinked PI TFC membranes testing performance.

Membrane	Composition	Pressure (bar)	Jsw (lmh)	Rob (%)	A (lmh/bar)	B (lmh)
TFC 1	16% PI 3:1 DI bath Non-xlinking	60	41.52	69.92	1.54	14.54
		120	28.73	68.85	1.22	11.10
		200	22.61	44.53	0.89	24.62
TFC 2	18% PI 3:1 DI bath Non-xlinking	60	21.25	87.26	0.75	2.72
		120	11.51	77.90	0.45	1.53
		200	8.47	58.65	0.31	5.50
TFC 3	20% PI 3:1 DI bath Non-xlinking	60	17.68	87.33	0.62	2.28
		120	11.10	81.62	0.40	2.28
		200	6.41	67.25	0.29	2.90
TFC 4	16% PI 3:1 IPA bath Non-xlinking	60	33.30	34.91	1.12	52.21
		120	22.40	36.27	0.87	36.56
		200	18.09	33.82	0.72	31.47
TFC 5	18% PI 3:1 IPA bath	60	13.56	46.36	0.45	14.18
		120	8.84	48.72	0.34	8.55

	Non-xlinking	200	6.78	56.77	0.26	4.79
TFC 6	20% PI<br>3:1<br>IPA bath<br>Non-xlinking	60	6.17	84.93	0.21	1.02
		120	4.77	77.47	0.16	1.29
		200	3.78	62.15	0.14	2.16
TFC 7	16% PI<br>1:1<br>DI bath<br>Non-xlinking	60	16.03	51.79	0.53	13.36
		120	9.82	35.98	0.33	16.02
		200	7.40	44.54	0.28	8.52
TFC 8	18% PI<br>1:1<br>DI bath<br>Non-xlinking	60	29.72	52.27	1.01	23.10
		120	24.01	51.71	0.94	19.49
		200	34.94	26.05	1.27	82.95
TFC 9	20% PI<br>1:1<br>DI bath<br>Non-xlinking	60	31.24	39.17	1.08	41.10
		120	21.21	22.27	0.79	65.11
		200	12.50	23.30	0.46	37.34
TFC 10	16% PI<br>1:1<br>IPA bath<br>Non-xlinking	60	58.21	50.21	2.07	44.23
		120	30.83	31.90	1.11	55.86
		200	19.53	26.02	0.68	49.12

TFC 11	18% PI<br>1:1<br>IPA bath<br>Non-xlinking	60	3.82	99.32	0.12	0.02
		120	2.92	86.46	0.09	0.43
		200	2.38	65.50	0.07	1.18
TFC 12	20% PI<br>1:1<br>IPA bath<br>Non-xlinking	60	5.96	13.29	0.19	36.16
		120	5.75	47.46	0.19	5.93
		200	4.11	46.76	0.11	4.38

Table S5. Tuned-TFX testing performance.

Membrane	Composition	Pressure (bar)	Jsw (lmh)	Rob (%)	A (lmh/bar)	B (lmh)
t-TFX	16% PI 3:1 IPA bath *Ex situ* *Tuned IP*	60	20.14	98.11	0.73	0.35
		120	15.62	98.79	0.69	0.18
		200	10.07	99.02	0.65	0.09
Commercial Membrane	Dupont HPRO	60	32.06	99.21	1.26	0.22
		120	18.91	99.32	0.95	0.12
		200	9.87	94.98	0.40	0.49
Handcast TFC membrane	Dlamini, D. S. et al.[32]	60	27.54	98.28	1.01	0.42
		120	13.56	96.31	0.53	0.48
		200	8.22	94.40	0.33	0.46

Table S6. Membrane characteristics.

Sample	Tg (°C)	XPS surface elemental analysis					Tensile test	Contact angle
		O (%)	N (%)	C (%)	O/N ratio	Crosslinking Degree (%)	Tensile Strength (MPa)	
PI	313						3.51	
PSU	181						6.31	
i-TFX	323	11.42	79.50	9.08	1.26	65.49	14.65	57.3
e-TFX	323	10.88	8.81	80.31	1.23	69.06	19.46	56.8
t-TFX	323	12.66	10.81	76.53	1.17	76.35	15.20	53.1
Commercial HPRO	181	8.33	84.62	7.05	1.18	75.23	3.82	47.4
