# Supplementary material for: Ultrahigh pressure compaction-resistant thin film crosslinked composite reverse osmosis membranes
Source: Nat Commun. 2025 Sep 1;16:8165. doi: 10.1038/s41467-025-63639-0 (PMC12402197; doi:10.1038/s41467-025-63639-0)
Supplement: Supplementary file 3 — Description of Additional Supplementary Files [file 41467_2025_63639_MOESM3_ESM.pdf]

Description of Additional Supplementary Files

Supplementary Movie 1.
In operando SEM of TFX UHPRO membrane.

Supplementary Movie 2.
In operando SEM of TFC HPRO membrane.

Supplementary Movie 3.
In operando SEM segmentation of TFX membrane.

Supplementary Movie 4.
In operando SEM segmentation of commercial TFC-HPRO membrane.
